# Urinary bio-monitoring of aromatic amine derivatives by new needle trap device packed with the multi-component adsorbent

**DOI:** 10.1038/s41598-023-31108-7

**Published:** 2023-03-14

**Authors:** Razzagh Rahimpoor, Khaled Murtada, Ali Firoozichahak, Babak Pashaei, Danial Soleymani-ghoozhdi, Houman Serkan, Faeze Mehregan, Saber Alizadeh

**Affiliations:** 1 Department of Occupational Health Engineering, Research Center for Health Sciences, School of Health, Larestan University of Medical Sciences, Larestan, Iran; 2 Department of Chemistry, University of Waterloo, 200 University Avenue West, Waterloo, ON N2L 3G1 Canada; 3 Department of Occupational Health, Faculty of Health, Social Determinants of Health Research Center, Gonabad University of Medical Science, Gonabad, Iran; 4 University of Mazandaran, Babolsar, Iran; 5 Faculty of Public Health, Kerman University of Medical Sciences, Kerman, Iran; 6 The Islamic Azad University, Science and Research Branch, Tehran, Iran; 7 School of Medicine, Shahrekord University of Medical Sciences, Shahrekord, Iran; 8 Department of Chemistry, Bu-Ali-Sina University, Hamedan, Iran

**Keywords:** Analytical biochemistry, Environmental chemistry, Biomarkers, Health occupations

## Abstract

Aromatic amines are a large group of chemical compounds that have attracted the attention of researchers due to their toxicity and carcinogenicity. This study aimed to develop an efficient method for sampling and analysis of aromatic amines (Aniline, N, N-dimethylaniline, 2-chloroaniline, and 3-chloroaniline) from the vapour phase (headspace) of urine samples. For the implementation of this plan, a needle trap device packed with the three-component adsorbent consisting of nano-Hydroxy Apatite (nHA), Zeolite (Ze), and Metal–Organic Framework (MOF) equipped with GC-FID was employed for the first phase. Examination of the prepared adsorbents was performed by FT-IR, PXRD, and FE-SEM techniques. The optimal value of considerable parameters such as time and temperature of extraction, salt content, and pH were established using the Response Surface Methodology-Central Composite Design (RMS-CCD) method. In this way, the optimal extraction of targeted analytes was accomplished in 41 min at 41 °C with NaCl content of 33.0% (w/v) and pH: 13.0, respectively. Also, the repeatability and reproducibility of the method were calculated to be in the range of 2.2–7.1% and 3.9–8.1%, respectively, which indicates the acceptable precision of the method. Also, the limit of detection (LOD) and limit of quantification (LOQ) were determined in the range of 0.3–32.0 ng.L^−1^ and 0.8–350.0 ng.L^−1^, respectively, which proves the high sensitivity of the proposed method. Furthermore, the recovery percent of the extracted analytes was concluded in the range of 97.0–99.0% after 6 and 30 days of the sampling and storage at 25 °C and 4 °C, respectively. Finally, the designed procedure was employed in the analysis of the above-mentioned aromatic amines in the real urine samples. The achieved results illustrate that the three-component absorbent system (nHA;Ze;MOF@NTD) can be introduced as an efficient, fast-response, sensitive, and versatile procedure for trace analysis of the different aromatic amine compounds in public and occupational health.

## Introduction

Aromatic amines are semi-volatile environmental pollutants with widespread use in paint, pesticide, explosive, drug, and rubber industries^1,2^. The most common sources of exposure to aromatic amines are tobacco smoke, smoke from a diesel engine, pesticides, and hair color^1^. The International Agency for Research on Cancer (IARC) has classified Aniline as a potential carcinogen (Group 2A)^3^. Also, the European Union has placed aromatic amines on the priority list of 1 chemical due to their toxicity and high production rates^4^. Previous studies have reported that long-term exposure to aromatic amines is associated with an increased risk of bladder cancer, dermatitis, hemolysis, and acute methemoglobinemia^1,2^. These compounds can be penetrated through the skin, gastrointestinal tract, and respiratory tract, and eventually excreted freely or conjugated in the urine^1,2^. Therefore, environmental and biological monitoring of these compounds as a complement to occupational exposure assessment is essential due to the mentioned risks^5^. Many studies have been performed on the determination of these compounds in the metabolized and non-metabolized forms in biological fluids such as plasma, milk, and urine^6–9^. But, the evaluation of the non-metabolized form of chemicals is usually preferred due to specific biomarkers of non-metabolized forms and the probability presence of other metabolites from various substances in the urine^10–13^. On the other hand, the trace level of aromatic amines in the urine causes problems in the diagnosis and determination process and monitoring of these samples seems to be a cumbersome process in adopting a system for headspace sampling. Therefore, developing a versatile monitoring procedure for accurate sampling and enrichment in a single run before the chromatographic section is still necessary by modification of current methods^9,14^.

Aromatic amines are generally adsorbed onto a porous adsorbent where the sample collection system operates in an active or passive mode. Microextraction techniques, such as solid phase microextraction (SPME) and the use of a needle trap device (NTD) are relatively new in the determination of aromatic amines.

SPME and HS-SPME techniques have been used as simple, rapid, one-step, and solvent-free methods for the determination of different aromatic amines in urine^10,12^. However, these methods show disadvantages such as fiber fragility, limited adsorption capacity, and short lifespan^15^. The needle trap device (NTD) was introduced in 2001 as a micro-extraction technique^16^. In this technique which consists of a packed needle with a solid adsorbent, the targeted analytes can be penetrated in the air environment or the upper space of aqueous solutions (headspace) in a non-equilibrium mode^17^. This technique provides a solvent-free procedure in a one-step sampling of guest analytes along with the addressed problems such as needle fragility and limited absorption capacity. So, it is noteworthy that the NTD technique as an efficient and green and flexible procedure can be adopted with the various solid adsorbents as packing agents^15^. The literature survey indicates attractive documents that have been reported by using the NTD packed with the single-component adsorbent (Hydroxy Apatite, MIPs, MOFs, silica gel, …) for the micro-extraction of the various analytes in the urine samples^15,17–21^.

On the other hand, NTD packed with a single-component adsorbent cannot have suitable efficiency for the simultaneous extraction of different compounds^22^. The modified NTDs packed with the multi-component absorbers can be a new insight into the extraction and determination of analytes concerning the key parameters of adsorption/desorption such as surface area, adsorption capacity, selectivity, polarity, and functional groups^22–24^. Therefore, multi-bed adsorbents are preferred according to the polarity of the analytes used^22^.

Nano hydroxyapatite is a mineral that is widely used in biomedical materials, environment and chemical engineering. Among the unique properties of hydroxyapatite nanoparticles, we can mention their cheapness, excellent biocompatibility, good thermal stability, ion exchange capability, non-toxicity, and excellent absorption properties^25–29^. In various studies, nHA has been used to extract polycyclic aromatic hydrocarbons (PAHs), phthalate esters and also volatile organic compounds^21,30–33^.

Metal–organic frameworks (MOFs) are new porous structures introduced in the early 1990s by Rabson and Hoskins^34^. Due to the presence of the same units in their structure, they have regular and uniform holes, which has increased their selectivity^35^. Recently, there has been a growing interest in using MOFs with porous structures and high adsorption capacity as adsorbents in various techniques^36–38^.

Zeolites are part of the family of hydrated aluminosilicates of alkali metals with a crystalline structure. This adsorbent has been used in various studies due to its special spatial structure, high cation exchange capacity, chemical, thermal and physical stability, low price, no need for recycling, and abundant variety^39–41^.

According to the best of our knowledge, no study has been reported on the determination of the aromatic amines in urine using the NTD packed with multi-component adsorbents. For the implementation of this idea, an NTD packed with a three-component adsorbent consisting of nano-Hydroxyapatite (nHA), Zeolite (Ze), and Metal–Organic Framework (MOF) combined with gas chromatography (GC) utilized for sampling, extraction, and analysis of aniline, *N*, *N*-Dimethylaniline, 2-Chloroaniline, and 3-Chloroaniline from the upper space of urine samples in the laboratory and real human samples. Also, optimization of operational and instrumental factors affecting the extraction (temperature, time, salt percentage, and pH) and desorption (temperature and time) steps were performed by response surface methodology (RMS) and central composite design (CCD). To validate the proposed method, the limit of quantification (LOQ), the limit of detection (LOD), accuracy, veracity, maintainability, carryover, and efficiency were determined. Finally, the efficiency of the proposed method was evaluated in real urine samples. The achieved results indicated that the designed method can be employed as a versatile and efficient procedure for the determination of aromatic amine derivatives in different industries.

## Materials and methods

### Chemical and reagents

Aromatic amines including Aniline (99.0%), *N*, *N*-dimethylaniline (99.0%), 2-chloroaniline (99.0%), and 3-chloroaniline (99.0%) were purchased from Merck (Germany) Darmstadt, Germany. Faujasite-type zeolite ((Na_2_,Ca,Mg)_3.5_[Al_7_Si_17_O_48_]·32(H_2_O), Merck, 99.0%), Benzene-1,3,5-tricarboxylic acid (H_3_BTC, Merck, 95.0%), potassium nitrate (KNO_3_, Sigma-Aldrich, 99.0%), diammonium hydrogen phosphate ((NH_4_)_2_HPO_4_, Sigma-Aldrich, 99.0%), calcium chloride (CaCl_2_, Sigma-Aldrich, 98.0%), Ethanol (C_2_H_5_OH, Merck, 99.0%), Sodium hydroxide (NaOH, Merck 99.0%) utilized as received without any further purification. Pure nitrogen gas (99.0%) was obtained from Roham Gas Company of Tehran-Iran.

### Multi-component adsorbent synthesis procedures

#### Nano-hydroxyapatite synthesis procedure

The utilized nano-hydroxyapatite (nHA) in multi-component adsorbent was synthesized by the previously reported documents^42^. Briefly, 5.29 g (0.4 M) of (NH_4_)_2_HPO_4_ was dissolved in 100.0 ml of distilled water at room temperature (solution A). Also, 6.65 g (0.6 M) of CaCl_2_ was dissolved in 100.0 ml of distilled water at room temperature (solution B). Then, solution B was added dropwise to solution A under stirring to precipitation of white powder. The pH solution was adjusted in the range of 10.0–11.0 by NaOH solution (0.1 N). The obtained nano-hydroxyapatite powder was rinsed with distilled water for the removal of the residual raw materials. Finally, the achieved nano-hydroxyapatite powder was aged in the oven at 90 °C for 24 h for evaporation of the remained solvent.

#### HKUST-1 MOF synthesis procedure

The HKUST-1 or (Cu_3_(BTC)_2_) MOF was prepared based on the previously reported document^43,44^. In a typical synthesis of HKUST-1, 0.42 g (2.0 mmol) of ligand source (Benzene-1,3,5-tricarboxylic acid) and 0. 0.75 g (3.6 mmol) of cation source (copper nitrate) were dissolved in 7.5 ml of ethanol and 7.5 ml of deionized, respectively. Then, the cation source was added dropwise to the ligand source under stirring at room temperature. The prepared solution was heated in an autoclave at 130 °C for 12 h. Finally, the synthesized HKUST-1 MOFs were rinsed with ethanol and distilled water (3 * 15.0 ml) and were kept overnight in the oven (100 °C) for removal of the residual raw materials and solvents, respectively.

### Instrumentation

In this study, a spinal needle (No. 20, Kozan, Japan-Tokyo) and a low-flow sampling pump (222 series, SKC. USA) connected to NTD were used to sampling of aromatic amines from the upper urinary space. A magnetic hot plate (TAT-94–1062, Iran) was utilized to heat urine samples during sampling. A digital thermometer (Testoterm GmbH) was employed for continuous monitoring of the inside temperature of the sampling glass container. The samples were analyzed by gas chromatography (Varian CP-3800) equipped with a flame ionization detector (FID) with a capillary column (CP7462: 30 m × 0.25 mm). The injection temperature was adjusted in the range of 200–300 °C and the detector temperature was set in the range of 300 °C. The oven temperature was programmed as follows: The initial temperature of the oven was set at 80 °C for 5 min, then reached up to 180 °C with a rate of 5 °C.min^−1^, and remained at this temperature for 5 min. Characterization of the multi-component adsorbent was performed using the following instruments: Field emission-scanning electron microscopy (FE-SEM) images were recorded by a HITACHI S-4160 instrument. Powder X-ray diffraction (PXRD) patterns were prepared by an APD 2000 vital structures instrument in Bragg–Brentano mode (2θ − θ geometry; Cu Kα1) using a linear position sensitive detector (SAINT-GOBain). FT-IR spectra were obtained by a Pekin-Elmer GX FT-IR spectrometer.

### Preparation of NTD

**Figure 1:**
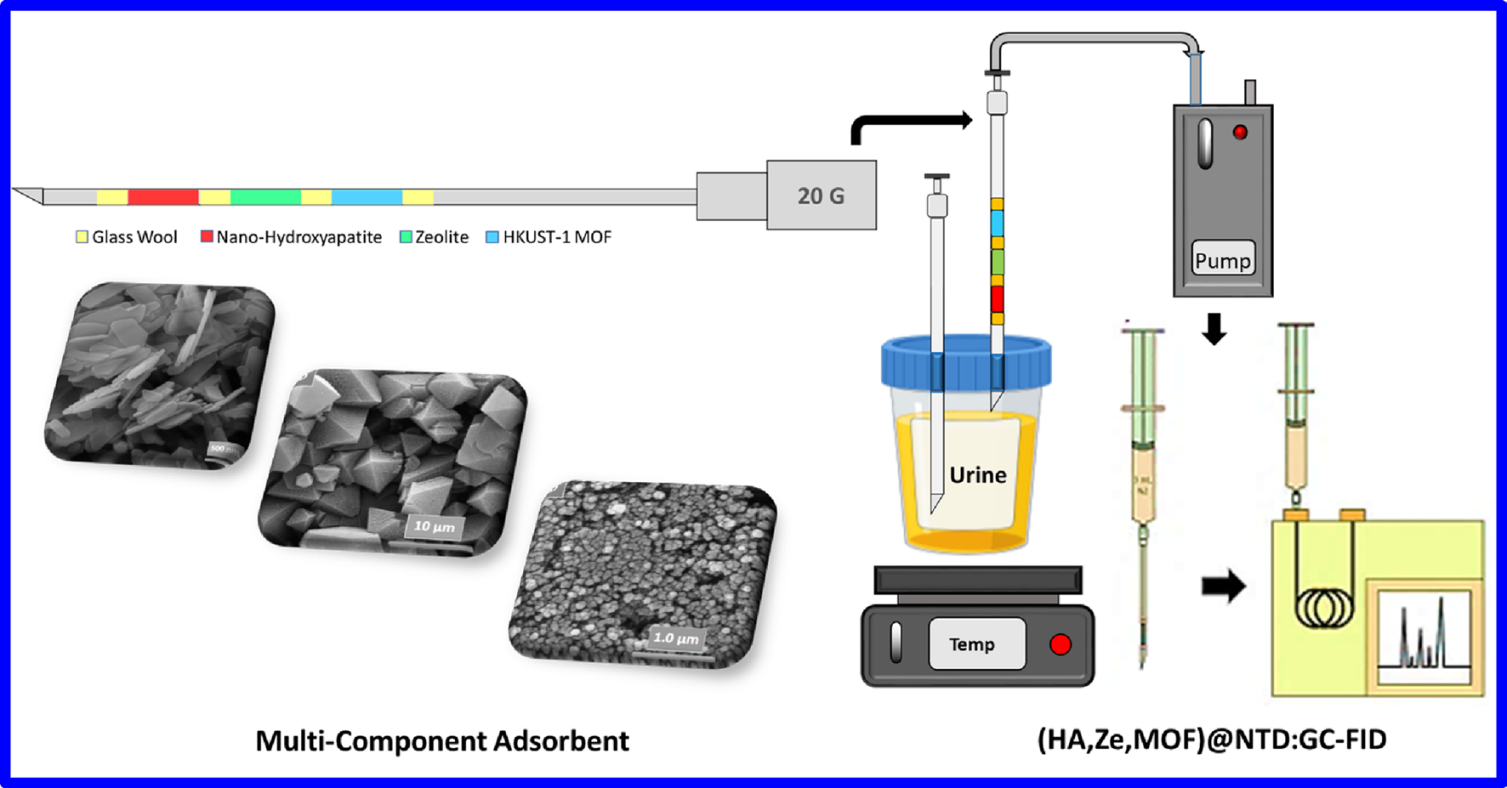
Graphical summary of sampling chamber and NTD packed with: nHA;Ze;MOF (Drawn by Dr. Saber Alizadeh).

A 20.0-gauge spinal needle was selected for the preparation of the NTD. Equal ratios of three adsorbents (HA: 2.0 mg, Ze: 2.0 mg, MOF: 2.0 mg) were packed into the needle (Fig. 1 and Figure S1). Both sides of the packed adsorbents were blocked by glass wool. A flow rate of 2.0 ± 1.0 mL.min^−1^ was selected by a soap bubble flow meter. The needles were placed at 300 °C at the injection site of the GC device for 2 h to condition and remove the disturbing factors from the intra-needle absorbers.

### Pilot study

Firstly, the standard stock solution (500.0 μg.mL^−1^) was prepared from the targeted analytes (Aniline, *N*,*N*-dimethylaniline, 2-chloroaniline, and 3-chloroaniline). Aromatic amine compounds were extracted by the HS-NTD activation method. According to Fig. 1 and Figure S1, a glass vial (20.0 ml) containing 10.0 ml of the desired solution at a concentration of 500.0 μg.mL^−1^ with different amounts of NaCl was prepared. The vial was hermetically sealed with a silicone-Teflon and aluminium cap to prevent the probable loss of targeted analytes from the upper space during extraction. The vial was heated indirectly using a water bath equipped with a thermometer. One side of the needles containing sorbents was placed in the sampling pump and the other side was placed in the upper part of the vial. To prevent of vacuum from forming inside the vial, another needle (Size: 20) was inserted into the vial fluid without an adsorbent. To evaluate the optimal temperature and time of desorption after extracting aromatic amines, the needles were placed at the injection site of the GC machine. Thermal desorption was performed by passing 3.0 ml of nitrogen gas using a Luer-lock syringe over the adsorbent.

### Response surface methodology

The response surface method (RSM) involves a set of mathematical and statistical techniques to model and analyze the problems in which the desired response is influenced by several variables. The first goal in RMS is finding the optimal response^45^. In the present study, the number of experiments was determined using the response surface method and central composite design (RSM-CCD).

### Extraction and desorption parameters

In this study, to evaluate the efficiency of (nHA;Ze;MOF)@NTD the simultaneous effect of several parameters such as temperature, time, salt, and pH was investigated in the range of 20–60 °C, 20–60 min, 0.0–40.0% and 7–13, respectively. These parameters were optimized by Design-Expert v7 (Stat Ease Inc., Minneapolis, USA) software. Also, the GC injection port was used for the desorption of aromatic amines. Desorption temperature and time were examined in the range of 200–300 °C and 1–6 min, respectively. Also, the interaction effect of these two parameters on desorption efficiency was evaluated. After drawing the calibration curves, the optimal desorption time was considered as the minimum time with the maximum area under the peak.

### Breakthrough volume (BTV)

The aim of the determination of BTV is to avoid egregious errors during analyte extraction. The BTV marks the limits of the sampling device’s capacity; as long as the BTV is not reached, sampling can continue. In this way, two similar NTDs were connected in series. After sampling under optimal conditions, the second NTD analytes were desorbed and the surface area under their peak was evaluated to determine the BTV.

### Carryover

The residual compounds in the adsorbent after desorption in terms of carryover effect can be leads to errors in subsequent extractions. To evaluate the carryover effect of the proposed method, the desorption process was performed by inserting NTD in the injection port of the GC device after sampling 500.0 μg.mL^−1^ (first desorption) under optimal conditions. The second desorption was repeated two min later and the NTD was inserted again into the injection port of the GC device. Then the obtained peak level of the second desorption was compared with the first desorption.

#### Storage time

To determine the storage time, both sides of the needles were sealed with paraffin after sampling of 500.0 μg.mL^−1^ by the proposed NTD under optimal conditions. The storage time of the studied analytes in the adsorbent was evaluated 1–6 days after sampling at 25 °C and 1–30 days after sampling at 4 °C.

#### Extraction efficiency

To evaluate the extraction efficiency (EF %) of the proposed method, firstly five different concentrations of analytes (10.0, 25.0, 50.0, 85.0, and 100.0 ng.L^−1^) were prepared in the urine matrix. Then, the extraction efficiency of aromatic amines was calculated according to the following formula:

$$Extraction \,efficiency = \frac{Meseared \,Concentration}{{Initial \,Concentration}} x 100$$

#### Enrichment factor

The enrichment factor (EF) of aromatic amines was obtained according to the slope calibration curve of the studied analytes after pre-concentration per slope calibration curve of direct injection of a standard solution of target analyte into GC- FID system. The enrichment factor for aromatic amines was calculated at the concentrations of 10–100 ng.L^−1^ by following Equation.

$$EF = \frac{{C_{F} }}{{C_{I} }}$$

where C_F_ and C_I_ are the concentration of aromatic amines after extraction and initial sample (10.0–100.0 ng.L^−1^).

#### Real sampling

To evaluate the performance of the proposed method in the field, the urinary upper space samples of 30.0 non-smoking workers in the plastic industry were examined at the end of the shift work under optimal conditions.

Urine samples were collected in polyethylene bottles and due to the volatility of the studied analytes, urine samples were analyzed immediately after sampling.

All methods carried out in accordance with the relevant guidelines and regulations of the declaration of Helsinki on medical research involving human subjects^46^. Also, all experimental protocols were performed according to the instructions of the General Medical Council and approved by the ethics committee of Gonabad University of Medical Sciences (IR.GMU.REC.1401.007). Informed consent was obtained from the participants of this study.

## Results and discussion

### Characterization of multi-component adsorbent

The characterization of the multi-component adsorbent was investigated by FT-IR, PXRD, and FE-SEM techniques.

**Figure 2:**
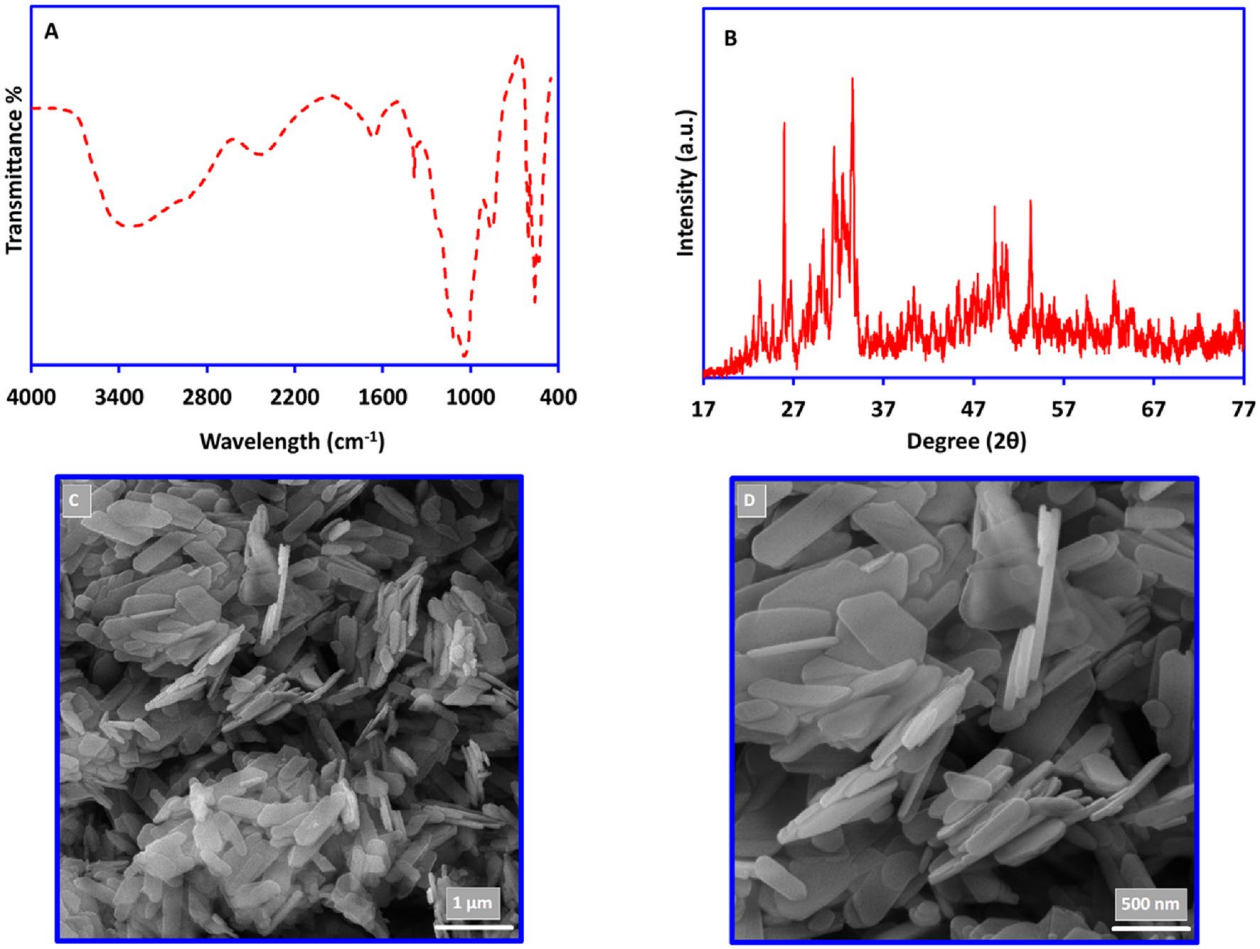
**(A)**: FT-IR, **(B):** PXRD, and **(C, D):** FE-SEM images of the synthesized nano-Hydroxy apatite (nHA).

**Figure 3:**
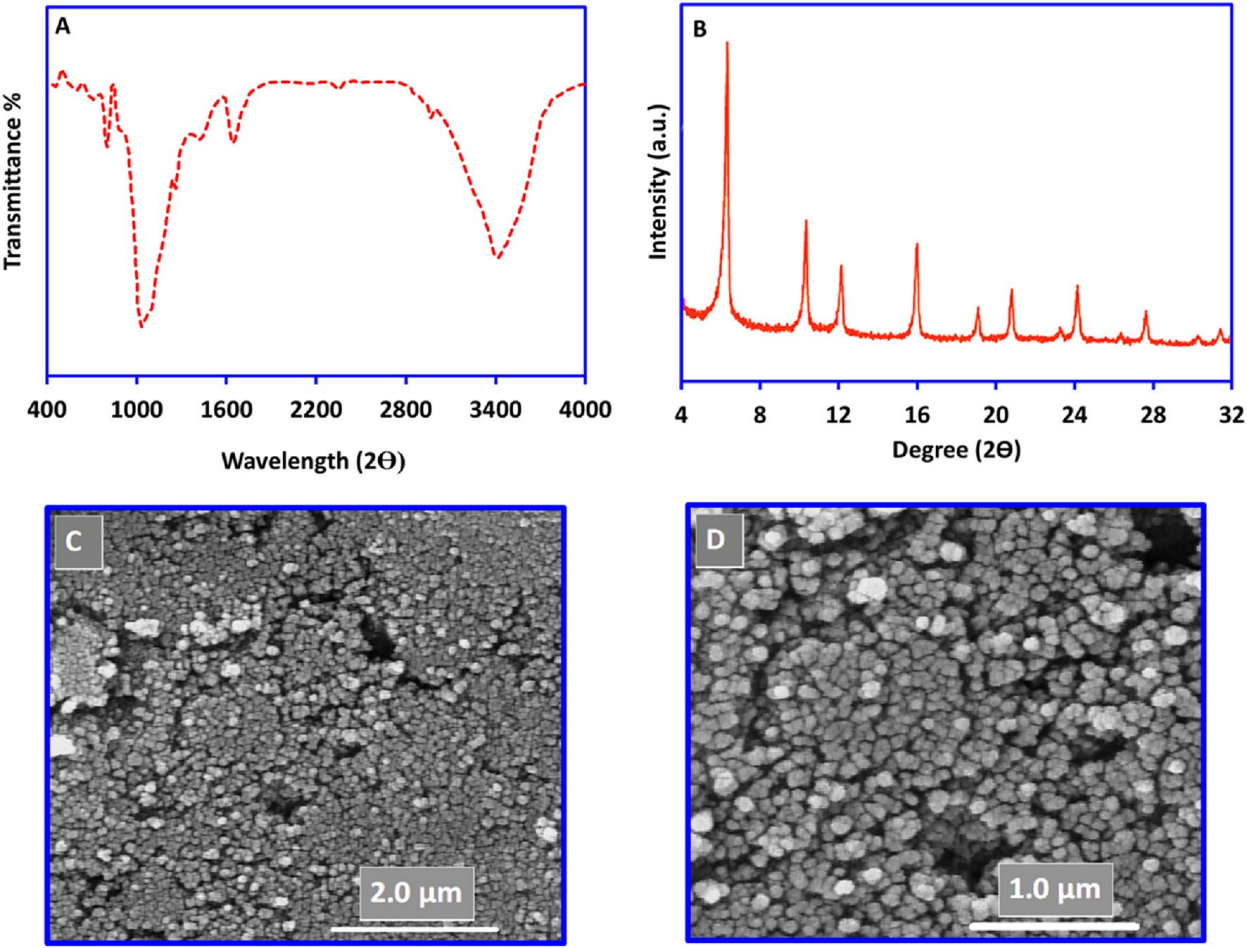
**(A)**: FT-IR, **(B)**: PXRD, and **(C, D)**: FE-SEM images of the commercial Zeolite (Ze).

**Figure 4:**
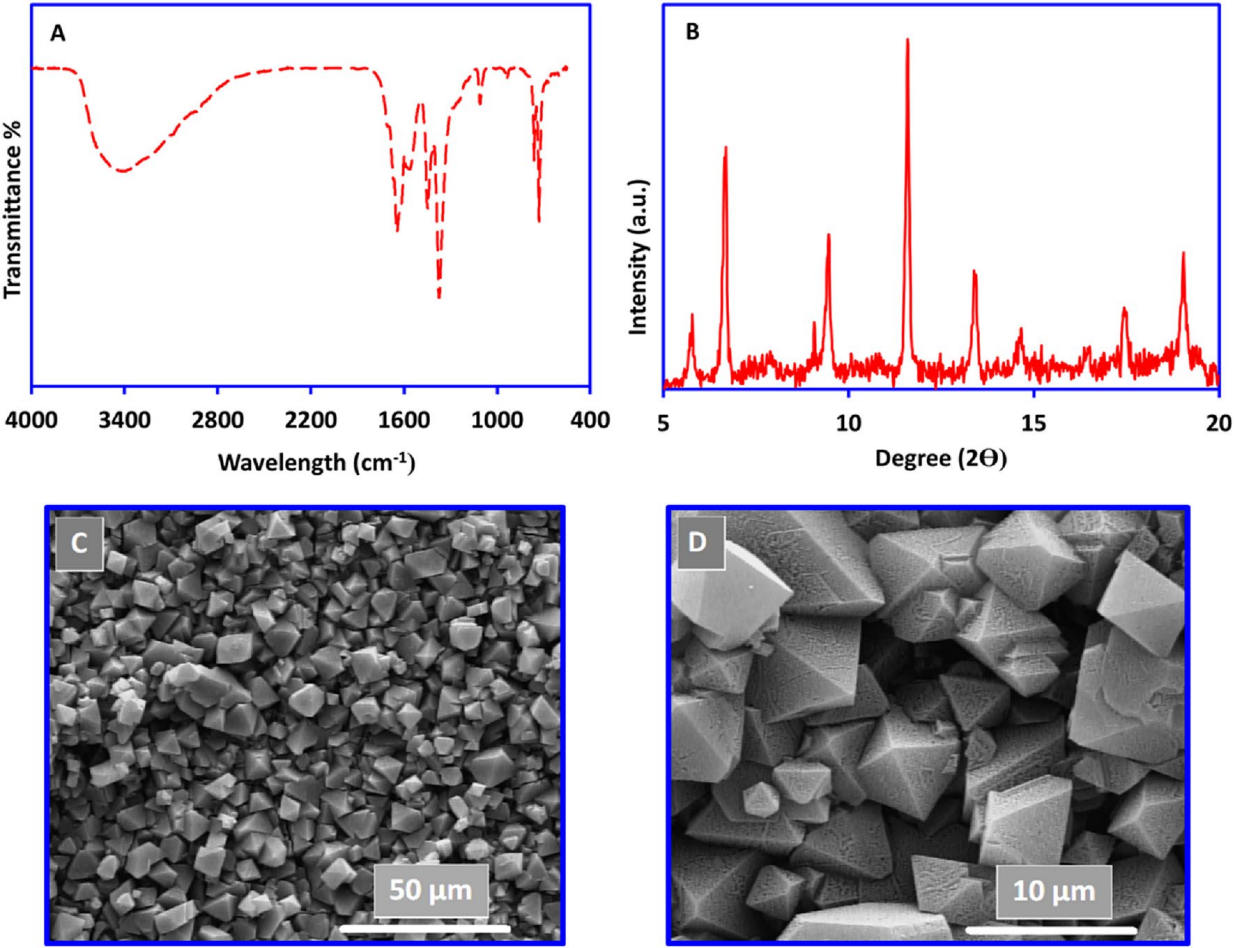
**(A)**: FT-IR, **(B)**: PXRD, and **(C, D)**: FE-SEM images of the synthesized HKUST-1 (MOF).

To confirm the bonding and functional groups in the employed multi-component adsorbent the FT-IR spectra of the HA, Ze, and MOF structures were recorded. Figure 2A shows the FT-IR spectrum of HA adsorbent. The related peaks to the phosphate bending vibration mode (567.0, 603.0 cm^−1^), phosphate stretching vibration mode (963.0 cm^−1^), and phosphate stretching vibration mode (1035.0, 1101.0 cm^−1^) can be shown in the pattern which is consistent with the previously reported document^29,44,47,48^. Figure 3A shows the FT-IR spectrum of commercial Ze adsorbent. This pattern shows bands at 1057.0 cm^−1^, 793.0 cm^−1^ and 467.0 cm^−1^ which can be assigned to internal vibrations of Si–O–Si and Si–O–Al bridges in the zeolite structure. Also, the appeared bands at 1637.0 cm^−1^ and 3200.0–3700.0 cm^−1^ can be related to the absorbed water by the zeolite. These observations can be approved by the previously reported documents^49,50^. Also, Fig. 4A shows the FT-IR spectrum of HKUST-1 adsorbent. According to the pattern, the absence of the broad carboxylic acid band (3085.0–2554.0 cm^−1^) can be related to the contribution of the above-mentioned groups in the HKUST-1 structure. Also, the asymmetric and symmetric vibration-coupled peaks of carboxylate anions (1646.0–1578.0 and 1443.0–1375.0 cm^−1^) can be related to the coordination of carboxylate groups with the Cu cations. Finally, the absence of free carbonyl groups at 1721.0 cm^−1^ can be approved for the coordination of the carbonyl with the Cu cations. These patterns and explanations are consistent with the previous related date^43,44,51^.

To investigate the purity and crystallinity of the multi-component adsorbent PXRD patterns of the HA, Ze, and MOF structures were recorded. Figure 2B indicates the X-ray diffraction pattern of the HA absorbent. The specific diffraction peaks can be assigned as hexagonal phases, consistent with the standard pattern of HA. The distinguished peaks at 2θ = 26.0, 31.7, 33.2, 35.2, 49.4 and 54.6 are consistent with the standard pattern of HA in the previously reported documents which can be assigned as hexagonal phase and suitable crystallinity of the synthesized nHA^29,44,47,48^. also, Fig. 3B indicates the X-ray diffraction pattern of the commercial NaY zeolite adsorbent. The appeared characteristic diffraction peaks at 2θ = of 6.0, 10.0, 12.0, 16.0, 19.0, 20.0, 24.0, 27.0, 31.0 and 32.0˚ can be assigned to the high crystallinity of zeolite Y structure without having any amorphous phase according to the reported documents^49,50^. Finally, Fig. 4B indicates the X-ray diffraction pattern of the HKUST-1 MOF adsorbent. The observed diffraction peaks at the 2θ = 5.8, 6.7, 9.5, 11.6, 13.4, 14.6, 16.4, 17.4, and 19.0 confirmed crystallization of the synthesized MOF consistent with the reported patterns^43,44,51^.

In the following, an FE-SEM image of the multi-component adsorbent was recorded to investigate its morphology. According to Figs. 2, 3 and 4C,D, HA, Ze, and HKUST-1 adsorbents consist of unique flake-like, thin polyhedrons, and blue sponge pyramidal crystals, respectively.

### Extraction parameters

**Figure 5:**
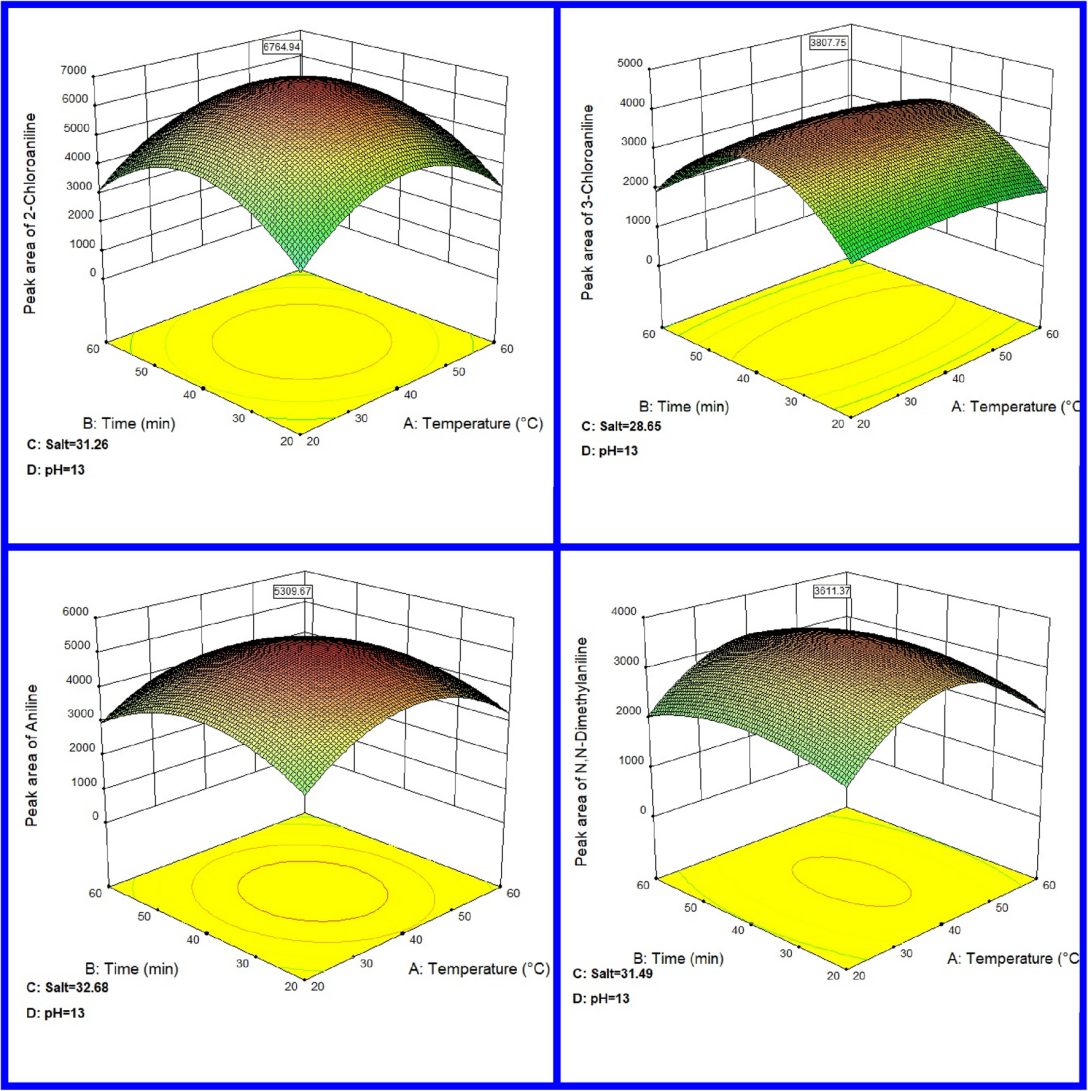
Optimization of extraction parameters of aromatic amine compounds sampled with nHA;Ze;MOF@NTD.

Extraction temperature can be affected by the partition coefficient of analytes in the gas–liquid and gas-adsorbent matrix. Increasing the extraction temperature is one of the ways to reduce the partition coefficient (K coefficient) and enhance the rate of analyte transfer from the liquid sample to the vapor upper space, which leads to an increase in extraction efficiency at a definite sampling time^52^. According to Fig. 5, the highest extraction efficiency of the analytes was determined at the range of 39–41 °C. But the extraction efficiency was decreased at the higher temperatures due to the interference of increased water molecules in the upper space with the absorption of analytes caused by increased vapour pressure at higher temperatures^20^. As a result, 41 °C was determined as the optimum temperature for all aromatic amines.

Extraction time is another factor that can be affected by the extracted analytes. At the shorter extraction times, the extraction efficiency can be decreased (because of the slower transferred rate of targeted analytes from the liquid matrix to the upper space of the vial) and high extraction time (the constant flow rate), can lead to a breakthrough. To optimize the extraction time, the proposed NTD was placed in the upper space of the sample for a specified time (20–60 min) and was immediately analyzed by GC. The highest extraction efficiency for the analytes was obtained in the time range of 38.44–41 min.

The presence of salt can reduce the solubility of analytes in the urine matrix and improves its transfer to the upper space of the vial^53^. To investigate the effect of salt presence on the extraction efficiency of aromatic amines, different amounts of NaCl from 0.0 to 40.0% (w/v) were added to the samples. The results showed that the highest extraction efficiency for analytes was obtained in the range of 28.0–33.0% (w/v). The extraction efficiency didn’t change with more NaCl contents. Therefore, 33.0% was selected as the optimal amount of salt to be added to the urine sample for the extraction of aromatic amines.

Aromatic amines as weak organic base compounds can be converted to simple molecules at a higher pH, which reduces their solubility in the sample. Hence, adjusting the pH of the solution is important^54^. In this study, the influence of pH was investigated by adding a strong base in the range of 7.0–13.0. The highest surface area under the peak was obtained at pH = 13.0. Table S2 shows the optimal levels of extraction parameters for urinary aromatic amines by the nHA;Ze;MOF@NTD method. High values of R^2^ and adj.R^2^ showed suitable adaptation of the model and dependence of the response variable on the input variables.

As a comparison, Sarafraz-Yazdi et al. have reported the HS-SPME: PEG/CNTs method to aromatic amine extraction from the upper urinary space. The optimal values of extraction temperature and time, salt content, and pH were determined to be 40 °C, 30 min, 30.0% (w/v), and 13.0, respectively^10^. Also, Youhong Ai et al. quantified aromatic amines in aqueous samples using the HS-SPME method. The optimal values of the above-mentioned parameters were appointed to be 40 °C, 40 min, 35.0%, and 11.0, respectively^54^. Furthermore, Abbasi et al. have used the PDMS-IL coated fiber for micro-extraction of the aromatic amines from the upper space of aqueous samples. The optimal values of extraction temperature, extraction time, and percentage of added salt were reported to be 30 °C, 40 min, and 35.0%, respectively^55^.

### Desorption parameters

**Figure 6:**
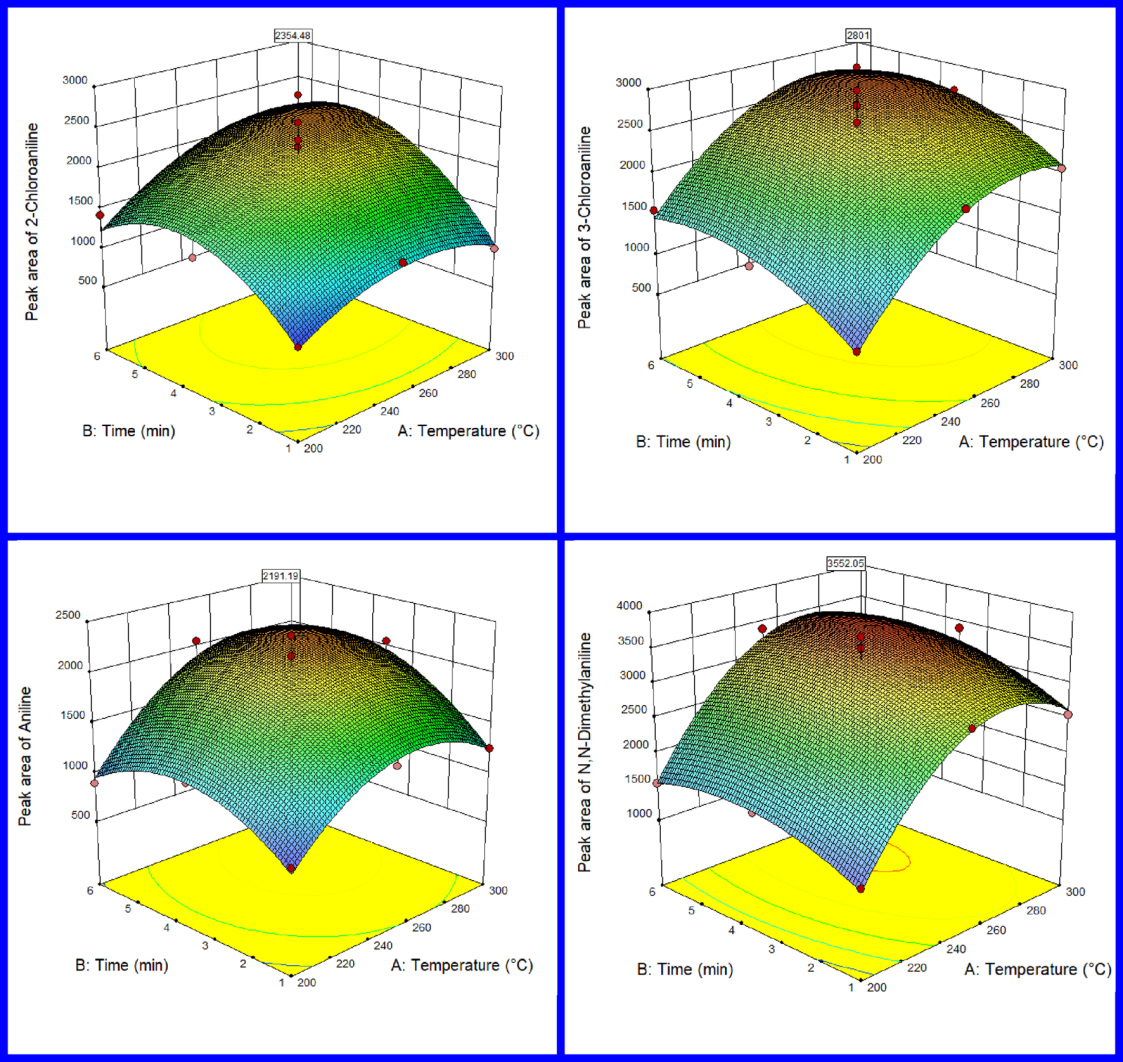
Optimization of desorption parameters of aromatic amine compounds sampled with nHA;Ze;MOF@NTD.

Thermal desorption is the method most commonly used to transfer analytes into analytical instrumentation. Complete desorption can improve the reproducibility, sensitivity, and reusability of the needles; therefore, it is essential to optimize the desorption process to ensure maximum efficiency. Furthermore, optimization of desorption time is required for the complete desorption of compounds of interest from the adsorbent bed. At lower than this time, desorption is incomplete and higher times are not necessary. The high temperatures and time may cause damage to the absorbent and decreases its life. Therefore, the distinguished effects of these two parameters (temperature and time of desorption) and their interaction on the sampling performance were investigated using the RSM-CCD method. According to the obtained data (Fig. 6), the optimal desorption temperature for the targeted analytes was gained in the range of 271–281 °C. Also, the optimal desorption time was considered to be 5 min. Table S1 shows the optimization results of the desorption parameters. The calculated R^2^ in this model is between 0.87 and 0.92 which indicates the appropriate response of the model. As a comparison, Sarafraz-Yazdi et al. have determined the optimum extraction temperature and time to be 280 °C and 20 s, respectively^10^. Also, these values were gained at 280 °C and 3 min in the Abbasi et al. study^55^.

### Breakthrough volume (BTV)

In this study, sampling of 50.0 µg. mL^-1^ was performed with a 2.0 mL/min flow rate. The sampling time was increased to 15 h to increase the sampling volume. According to the results, as time goes on, the surface area under the peak of the analytes increased. However, BTV was not observed even after 15 h of sampling with the proposed NTD which indicates the high absorption capacity of the proposed NTD.

### Carryover

In this study, the memory effect was not observed in any analytes at the optimum point (Desorption temperature = 281 °C and desorption time = 5 min). As a comparison, the Carryover effect in PANI as fiber in the HS-SPME system to the sampling of aromatic amines from wastewater reported by Minjia et al. was determined to be 250 °C and 10 min, respectively^56^.

### Storage time

**Figure 7:**
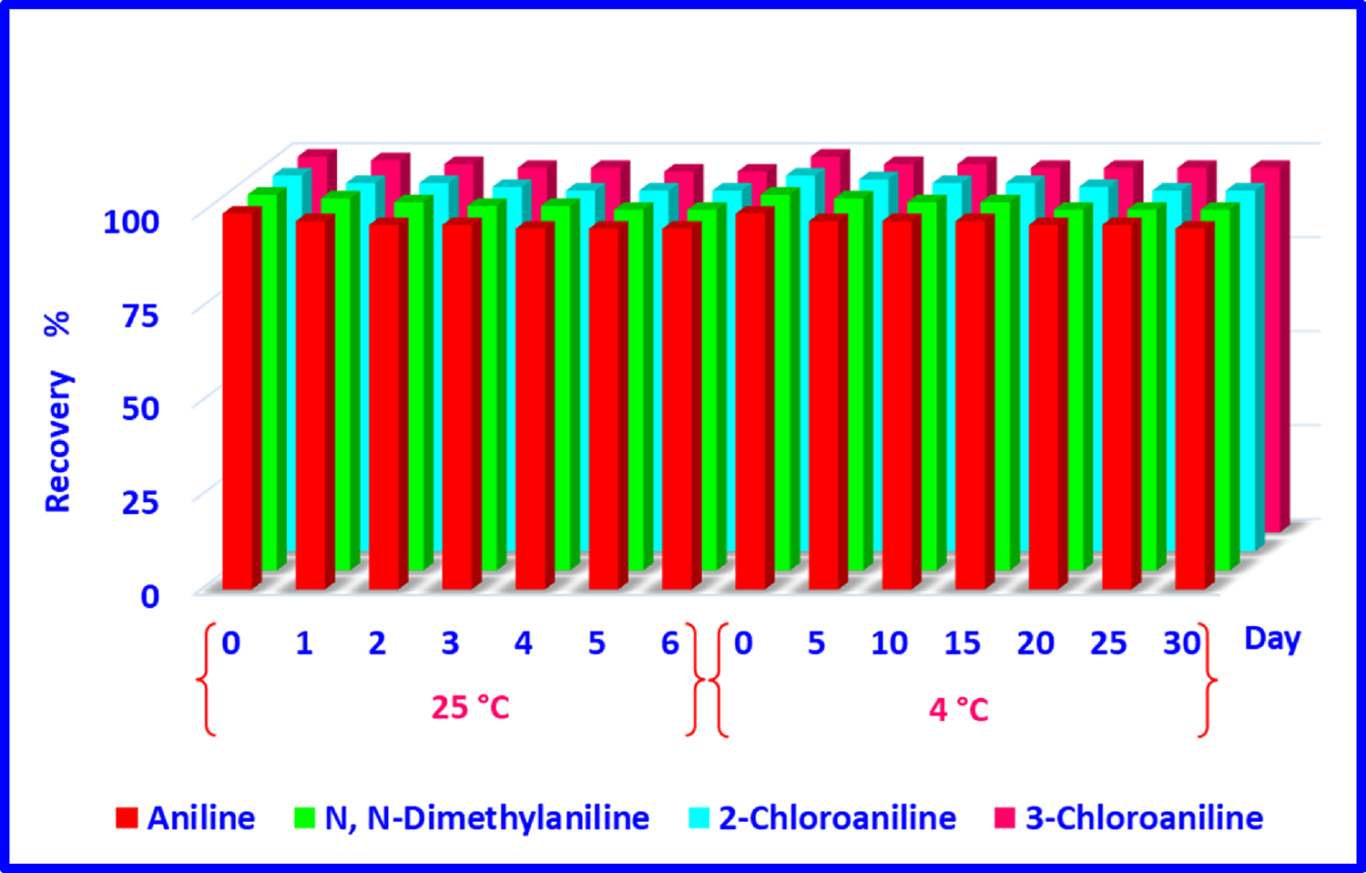
Storage times of aromatic amine compounds trapped by nHA;Ze;MOF@NTD from 1 to 30 days at 4 °C and 1 day to 6 days at 25 °C.

The results of the levels of analytes recovered on different days were compared to the sample analysis results immediately after sampling. According to the obtained results (Fig. 7), any significant change was not observed in the peak level of the studied analytes. As a result, the sampling of aromatic amines with the NTD packed with a three-component adsorbent can be stored for 6 and 30 days at 25 and 4 °C, respectively. It should be noted that NIOSH 2002 has suggested a storage period of more than 7 days for aniline at 25 °C^57^.

### .

#### Method validation

The validation method was performed by conforming to the International Union of Pure and Applied Chemistry (IUPAC) recommendations for determining and analyzing biological samples^58^. In this research, an external calibration curve was plotted for the range of 0.0008–800.0 × 10^7^ µg L^−1^. The linearity of the calibration curve was determined according to correlation coefficients (R^2^: 98–99).

Furthermore, using the optimal conditions, analytical figures of merit including linear range, limits of detection (LODs), limits of quantification (LOQs), and precision (% RSD) were evaluated to validate the developed method (Table 1). The linear range was determined up to 700.0 × 10^7^ ng L^−1^ of the target amines. LODs and LOQs calculated based on signal-to-noise ratios (SNR) of 3 and 10, respectively, were in the range of 0.3–32.0 µg mL^−1^, and 0.8–350.0 µg mL^−1^, respectively.

**Table 1:** The LOD and LOQ values for distinguished adsorbents. The repeatability of nHA;Ze;MOF@NTD for extraction of aromatic amines compounds.

Analytes	LOD (ng L^−1^)			LOQ (ng L^−1^)			Intra-day RSD% at different concentrations (ng L^−1^)					Inter-day RSD% at different concentrations (ng L^−1^)				
	MOF	Zeolite	nHA	MOF	Zeolite	nHA	10	25	50	85	100	10	25	50	85	100
Aniline	0.7	0.8	0.7	5.0	9.0	5.0	5.3	3.3	2.9	5.9	4.8	4.2	5.7	6.2	5.7	5.5
*N*, *N*-Dimethylaniline	0.7	0.8	0.7	5.0	9.0	5.0	2.8	4.2	5.1	3.8	4.7	3.8	6.4	5.4	4.9	6.5
2-Chloroaniline	10.0	14.0	10.0	80.0	95.0	80.0	3.3	4.7	2.8	2.2	5.3	5.9	5.3	7.1	4.7	3.6
3-Chloroaniline	85.0	100.0	85.0	850.0	1000.0	850.0	4.1	3.6	3.4	3.7	4.8	6.1	4.9	3.9	5.8	4.8

The lower limit of quantification (LLOQ) for aniline, Dimethylaniline, 2-Chloroaniline and 3-Chloroaniline was estimated 0.9, 0.9, 9.0 and 360 ng.L^−1^, respectively.

Also, The upper limit of quantification (ULOQ) for aniline, Dimethylaniline, 2-Chloroaniline and 3-Chloroaniline was estimated 100.0, 100.0, 210.0 and 600.0 ng.L^−1^, respectively.

Intra- and Inter-day precision established by performing five consecutive extractions with one NTD, were in the range of 2.8–5.3% and 3.6–7.1%, respectively, at five different concentrations (10.0, 25.0, 50.0, 85.0, 100.0 µg mL^−1^) for all target amines. The reproducibility of the method determined by sampling three NTD devices with the same characteristics was in the range of 3.9–8.1% at the concentration of 50.0 µg mL^−1^.

DeBruin et al.^7^ showed that the precision values for the HS-SPME sampling of aniline and 2-chloroaniline in urine matrix, were in the range of 2.7–8.5%. In another study, Sarafraz-Yazdi et al.^10^ reported that the repeatability of the HS-SPME for the analysis of monocyclic aromatic amines, were in the range of 5.1–9.1%.

### Extraction efficiency

As can be seen in Table 2, the results indicate that the extraction efficiency of aromatic amines from the upper space of urine was in the range of 97.0 to 99.0%, which is comparable to the other reported studies. Sarafraz-Yazdi et al.^10^ reported the extraction efficiency of monocyclic aromatic amines from real urine, was in the range of 63.7–79.1%. The extraction efficiency and enrichment factor of the aromatic amines demonstrate that the extraction efficiency results of the current method are acceptable.

**Table 2:** Investigation of reproducibility, recovery percentage, and enrichment factor of aromatic amine compounds sampled with nHA;Ze;MOF@NTD.

Analytes	RSD% for different NTDs at concentration of 50.0 ng L^−1^			Recovery% at different concentrations ( ng L^−1^)					Enrichmet factor				
	NTD1	NTD2	NTD3	10.0	25.0	50.0	85.0	100.0	10.0	25.0	50.0	85.0	100.0
Aniline	5.9	7.2	3.9	98.0	97.0	99.0	97.0	99.0	28.4	29.2	30.2	31.2	32.5
Dimethylaniline	6.3	4.3	7.8	99.0	98.0	97.0	98.0	98.0	29.5	31.5	32.8	34.2	33.5
2-Chloroaniline	4.8	3.9	8.1	97.0	99.0	98.0	99.0	98.0	27.4	28.1	28.6	29.5	30.8
3-Chloroaniline	4.9	5.2	7.9	98.0	97.0	98.0	97.0	97.0	28.2	29.0	29.8	31.7	32.4

### Real sample

The established method was applied to analyze real urine samples (nonsmokers) for the determination of the contents of aromatic amines, and the results are shown in Table 3. Based on the presented results in Table 3, the concentration of aniline in real urine samples extracted by using nHA;Ze;MOF@NTD was estimated in the range of 18.0–32.0 µg.mL^−1^. It should be noted, the studied population had an average age and work experience of 42.0 ± 0.8 and 12.0 ± 0.3 respectively. Also, all the studied subjects were male.

**Table 3:** The concentration of aniline in urine samples of workers in the plastic industry.

Sample	Concentration of aniline (µg.mL^−1^)	RSD %	Sample	Concentration of aniline (µg.mL^−1^)	RSD %
Sample 1	23.0	5.6	Sample 16	31.0	5.5
Sample 2	29.0	6.9	Sample 17	21.0	4.9
Sample 3	26.0	7.8	Sample 18	22.0	4.9
Sample 4	18.0	5.9	Sample 19	29.0	8.7
Sample 5	31.0	6.8	Sample 20	26.0	7.2
Sample 6	24.0	5.2	Sample 21	20.0	6.9
Sample 7	32.0	9.1	Sample 22	27.0	9.1
Sample 8	29.0	4.3	Sample 23	19.0	5.7
Sample 9	26.0	6.1	Sample 24	21.0	6.3
Sample 10	24.0	7.3	Sample 25	22.0	5.0
Sample 11	22.0	7.1	Sample 26	20.0	7.2
Sample 12	23.0	6.0	Sample 27	21.0	9.5
Sample 13	21.0	4.8	Sample 28	33.0	7.9
Sample 14	19.0	9.1	Sample 29	28.0	5.3
Sample 15	27.0	7.1	Sample 30	27.0	6.8

Figure S2 and S3 also show the obtained chromatograms from the extraction and analysis of aromatic amines in the headspace of urine samples using the nHA;Ze;MOF@NTD technique.

Finally, various analytical parameters such as linearity ranges, LOQ and LOD values obtained from the proposed method, were compared with previously published reports for the analysis of aromatic amine compounds (Table 4). As can be seen in Table 4, the proposed method has lower LOD and LOQ values than other studies, which indicates the high sensitivity of the method. Also, the proposed method has LDR up to 700.0 ng.L^−1^, which indicates a high adsorption capacity that can be used for environments with higher concentrations. Therefore, nHA;Ze;MOF@NTD can be used as a sensitive, environmentally friendly, user-friendly, and cost-effective method to determine low levels of aromatic amines in the urine matrix.

**Table 4:** Comparison of nHA;Ze;MOF@NTD with other methods for sampling and analysis of aromatic amine compounds.

Methods	Current method (ng. L^−1^)			HS-SPME/PEG@CNTs (ng. L^−1^)^14^			HS-SPME/PDMS-IL (ng. mL^−1^)1^43^			HS-SPME/HIL-doped PANI (µg.L^−1^)^42^			HS-SPME/PANI (µg.ml^−1^)^44^		
Parameters	LOD	LOQ	LDR (107)	LOD	LOQ	LDR (105)	LOD	LOQ	LDR	LOD	LOQ	LDR	LOD (10–3)	LOQ	LDR
*Comparison*															
Aniline	0.3	0.8	700	0.5	1	1	0.01	0.05	0.05–500	0.024	NR	0.195–100	0.69	NR	0.069–27.5
Dimethylaniline	0.3	0.8	700	0.5	1	1	0.001	0.005	0.005–500	NR	NR	NR	1.06	NR	0.053–21.3
2-Chloroaniline	3	8	700	5	50	50	0.1	0.5	0.5–500	0.012	NR	0.097–50	0.02	NR	0.0051–20.4
3-Chloroaniline	32	350	700	50	500	500	0.1	0.5	0.5–500	0.048	NR	0.390–100	NR	NR	NR

One of the limitations of the study is the pressure drop caused by packing three absorbers in the needle, so it is suggested to use larger gauges of the needle in future studies.

## Conclusion

In this study, the non-metabolized form of aromatic amines was successfully extracted from the upper space of urine samples for the first time by using a needle packed with the multi-component adsorbent (nHA, Ze, and MOF). The extraction and desorption parameters of the process were optimized by using the response surface methodology and central composite design (RSM-CCD). The results indicated that the proposed NTD has a high BTV and high extraction efficiency. Therefore, it has a good adsorption capacity for aromatic amines. The needle packed with three absorbents equipped with the GC-FID provided a low detection limit, high sensitivity, and wide linear dynamic range. Finally, this method can be employed as a simple, and solvent-free method to extract and determine the trace level of aromatic amines in the headspace of urine samples. Furthermore, this method can also be applied to the analysis of heterocyclic aromatic amines from food samples; as such, as a future direction, we aim to validate the method for the determination of heterocyclic aromatic amines in foods. As well, the feasibility of the method for analysis of volatile organic compounds in different types of samples is under consideration.

## Supplementary Information

Supplementary Information.

## Data Availability

The datasets used and analysed during the current study are available from the corresponding author on reasonable request.
