# Supplementary material for: Urinary bio-monitoring of aromatic amine derivatives by new needle trap device packed with the multi-component adsorbent
Source: Sci Rep. 2023 Mar 14;13:4243. doi: 10.1038/s41598-023-31108-7 (PMC10014860; doi:10.1038/s41598-023-31108-7)
Supplement: Supplementary file 1 — Supplementary Information. [file 41598_2023_31108_MOESM1_ESM.docx]

# Urinary Bio-Monitoring of Aromatic Amine Derivatives by New Needle Trap Device Packed with the Multi-Component Adsorbent

Razzagh Rahimpoor^1^, Khaled Murtada^2^, Ali Firoozichahak^*3^, Babak Pashaei^4^, Danial soleymani-ghoozhdi^5^, Houman Serkan^6^, Faeze Mehregan^7^, Saber Alizadeh^8^

*^1^Department of Occupational Health Engineering, Research Center for Health Sciences, School of Health, Larestan University of Medical Sciences, Larestan, Iran*

*^2^* *Department of Chemistry, University of Waterloo, 200 University Avenue West, Waterloo, ON, N2L 3G1, Canada.*

*^3^ Department of Occupational Health, Faculty of Health, Social Determinants of Health Research Center, Gonabad University of Medical Science, Gonabad, Iran*

*^4^ Assistant Professor of Inorganic Chemistry, University of Mazandaran*

*^5^* *Student Research Committee, Faculty of Public Health, Kerman University of Medical Sciences, Kerman, Iran*

*^6^* *The Islamic Azad University, Science and Research Branch, Tehran*

*^7^* *Medical student, school of medicine, Shahrekord University of Medical Sciences, Shahrekord, Iran*

*^8^Department of Chemistry, Bu-Ali-Sina University, Hamedan, Iran.*

| Table S1. Response Surface Quadratic desorption model of aromatic amines compounds by HA;Ze;MOF@NTD method | | | | |
| --- | --- | --- | --- | --- |
| 3-Chloroaniline | **2-Chloroaniline** | **N,N-Dimethylaniline** | **Aniline** | **Parameters/analytes** |
| 280.42 | 278.44 | 271.67 | 271.17 | Optimal Temperature (◦C) |
| 4.66 | 4.56 | 4.47 | 4.34 | Optimal Time (min) |
| 0.87 | 0.88 | 0.95 | 0.92 | R-Squared |
| 0.78 | 0.79 | 0.91 | 0.8631 | Adj R-Squared |
| 291.52 | 255.13 | 226.68 | 182.98 | SD |
| 13.37 | 14.36 | 8.12 | 10.84 | CV |
| 1.191E+006 | 2.246E+006 | 1.061E+006 | 7.613E+005 | PRESS |
| 0.10 | 1.26 | 0.36 | 0.7736 | Lack of Fit |
| 0.004 | 0.0040 | 0.0002 | 0.0010 | p-value |

| Table S2. Response Surface Quadratic extraction model of aromatic amines compounds by HA;Ze;MOF@NTD method | | | | |
| --- | --- | --- | --- | --- |
| 3-Chloroaniline | **2-Chloroaniline** | **N,N-Dimethylaniline** | **Aniline** | **Parameters/analytes** |
| 39.17 | 40.79 | 39.33 | 39.93 | Optimal Temperature (◦C) |
| 39.86 | 40.50 | 39.44 | 38.44 | Optimal Time (min) |
| 28.64 | 31.26 | 30.33 | 32.68 | Optimal Salt (%) |
| 13 | 13 | 13 | 13 | Optimal pH |
| 0.95 | 0.94 | 9.93 | 0.92 | R-Squared |
| 0.91 | 0.89 | 0.87 | 0.85 | Adj R-Squared |
| 319.91 | 574.32 | 392.77 | 455.57 | SD |
| 16.09 | 21.40 | 20.44 | 23.49 | CV |
| 4.123E+006 | 1.490E+007 | 3.290E+006 | 9.619E+006 | PRESS |
| 0.9459 | 0.5335 | 0.8345 | 0.7517 | Lack of Fit |
| < 0.0001 | < 0.0001 | < 0.0001 | < 0.0001 | p-value |


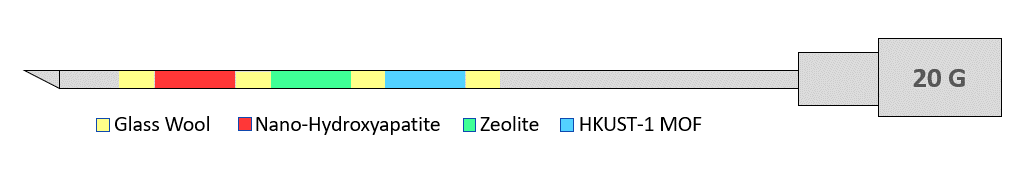


**Figure S1:** Schematic diagram of a needle trap device packed with the multi-component adsorbent (nHA;Ze;MOF)


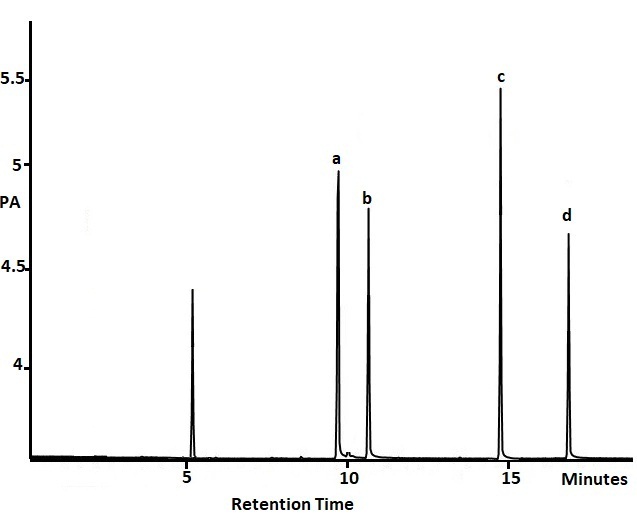


**Figure S2:** Chromatogram obtained from extraction and analysis of aromatic amines in the head space of urine samples by NTD: nHAP@silica gel@Perlite ; GC-FID technique. a: Aniline, b: N, N-dimethylaniline, c: 2-chloroaniline, d: 3-chloroaniline


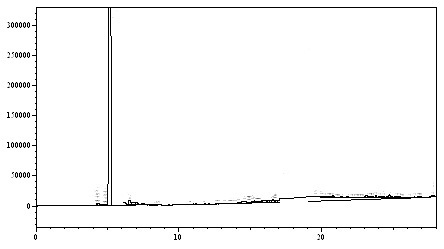


**Figure S3:** Chromatogram of Blank
